# First case of primary appendiceal tuberculosis presented as stump appendicitis

**DOI:** 10.1093/jscr/rjad373

**Published:** 2023-06-27

**Authors:** Midhun Mathew, Tony Mathew, Elizabeth Joseph

**Affiliations:** Masters of Surgery in Surgical Science and Practice, Royal College of Surgeons in Ireland, Dublin 2 D02 YN77, Ireland; Department of General Surgery, Believers Church Medical College Hospital, Thiruvalla, Kerala 689103, India; Department of Pathology, Believers Church Medical College Hospital, Thiruvalla, Kerala 689103, India

**Keywords:** tuberculosis, stump appendicitis, extrapulmonary tuberculosis, gastrointestinal tuberculosis

## Abstract

Tuberculosis (TB) remains as a significant global public health issue, especially in developing and underdeveloped nations. Extrapulmonary TB comprises 20% of the TB cases; of which 34.4% were in the lymphatics, 25.2% pleural, followed by 12.8% gastrointestinal and 9.4% in the central nervous system. Ileocecal involvement is the most common among gastrointestinal TB. Although it can cause secondary damage to the appendix, primary type of appendicular TB is rare and can occur with no other signs of the disease. A high index of suspicion is necessary for early diagnosis and treatment of TB. Similarly, stump appendicitis (SA) is a rare and delayed complication of appendectomy. Here we report a case of primary appendicular TB in a patient presented to a multi-specialty hospital in Kerala, India, with SA.

## INTRODUCTION

The WHO’s global tuberculosis (TB) report 2022 states that the COVID-19 pandemic has interrupted, or reversed the progress made in the fight against TB until 2019 [1]. The report states that India accounted for 28% of the world’s two-thirds of TB infections, and 32% of all TB deaths in 2021. India also has a high prevalence of gastrointestinal TB, with the ileocecal region being the most often affected area (75% of the GI tuberculosis) [2, 3]. Remarkably, the appendix rarely becomes involved despite being so near to the ileocecal region, with isolated primary appendicular TB seen in only about 1.5 to 3% of cases [4]. Because of its low incidence and low frequency of reporting in the literature, appendicular TB is a less well-known condition [5]. Accurate diagnosis of TB appendix is also not possible as patients present with a clinical picture of that of acute appendicitis with no signs suggestive of tuberculosis infection of the organ. Therefore, histopathological examination aids in the diagnosis of this condition.

Stump appendicitis (SA) is a rare and delayed complication of appendectomy, where the residual appendicular stump is re-inflamed [6]. According to estimates, the lifetime risk of experiencing acute appendicitis is 7%, but the risk of developing SA is substantially lower (1/50000) [7]. A retrospective review from 2008 to 2017 conducted in Turkey reported the incidence rate of SA to be at 0.15% [8]. Despite its rarity, SA should be an important differential diagnosis for any patient who has had an appendectomy and is experiencing right lower quadrant abdominal pain. A delayed diagnosis may cause delayed treatment and an increase in morbidity with complications including perforation and peritonitis.

The peculiarity of our case was the finding of appendicular TB presented as SA. This may even be the first report with a combined occurrence of both the conditions.

## CASE PRESENTATION

**Figure 1:**
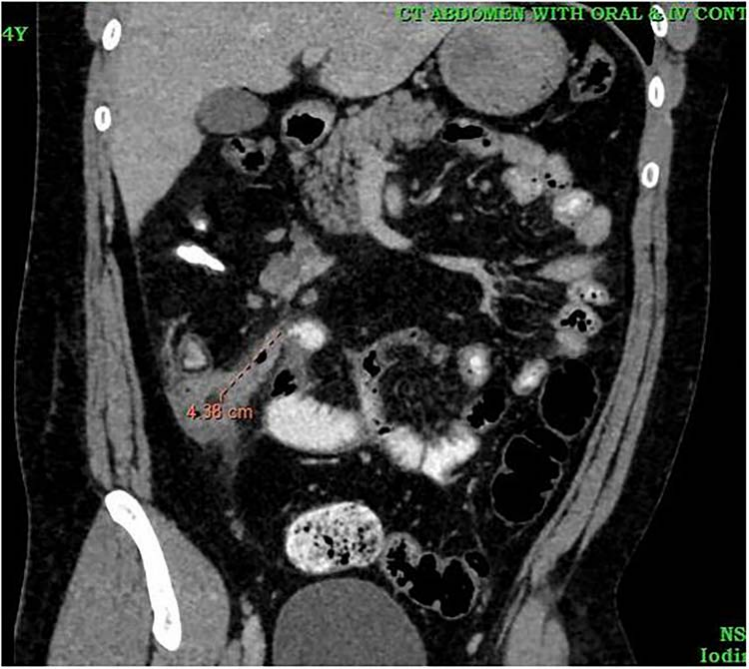
CT image showing inflammed stump appendix.

**Figure 2:**
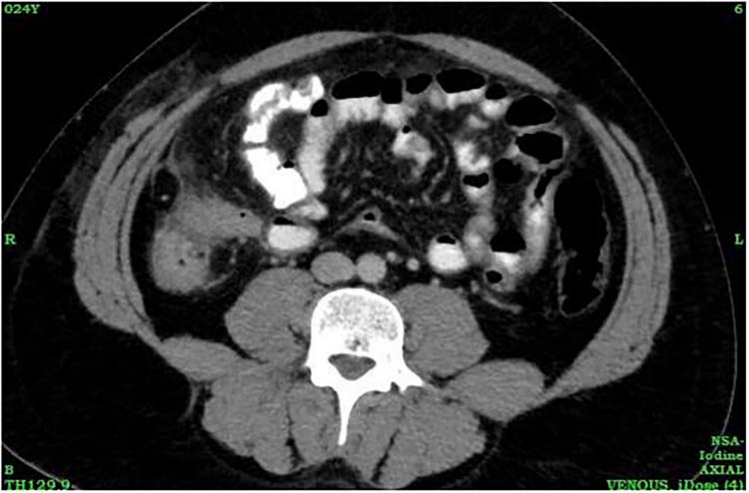
CT abdomen showing caecal wall thickening.

A 24-year-old male was admitted to the General Surgery out-patients clinic with a 2-week history of right iliac fossa pain. The patient had no other associated gastrointestinal symptoms. He had no other significant medical history but had undergone an open appendectomy for perforated appendicitis three months prior to the current presentation. Patient was vitally stable on presentation and the per abdominal examination showed tenderness at the McBurney’s point. A computed tomography (CT) scan of the abdomen revealed thickened and enhanced stump appendix along with adjacent fat stranding and multiple mesenteric nodes; suggestive of SA (Fig. 1). There was also a suggestion of adhesions and mild wall thickening of adjacent caecal wall with no evidence of collections or bowel mass (Fig. 2). Considering the upward trend seen in white blood cell count and the CT report, we planned the patient for a diagnostic laparoscopy. Pre-anaesthesia clearance was obtained.

**Figure 3:**
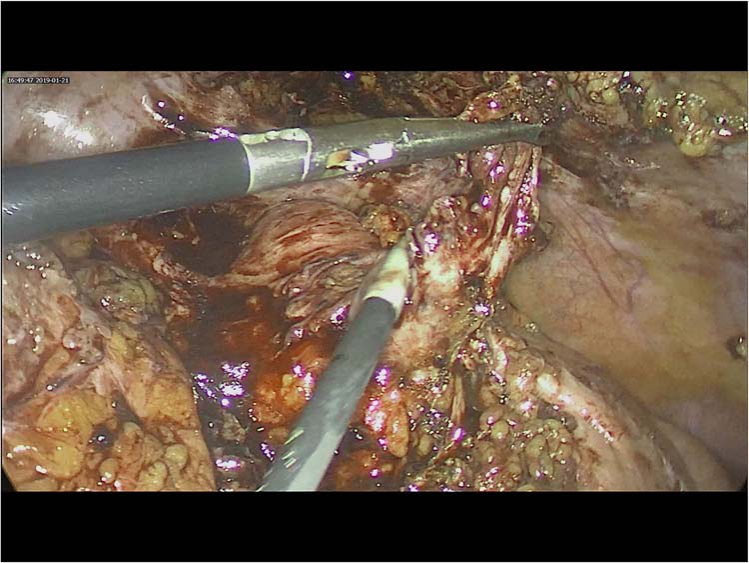
Intra-operative image of appendicular stump.

**Figure 4:**
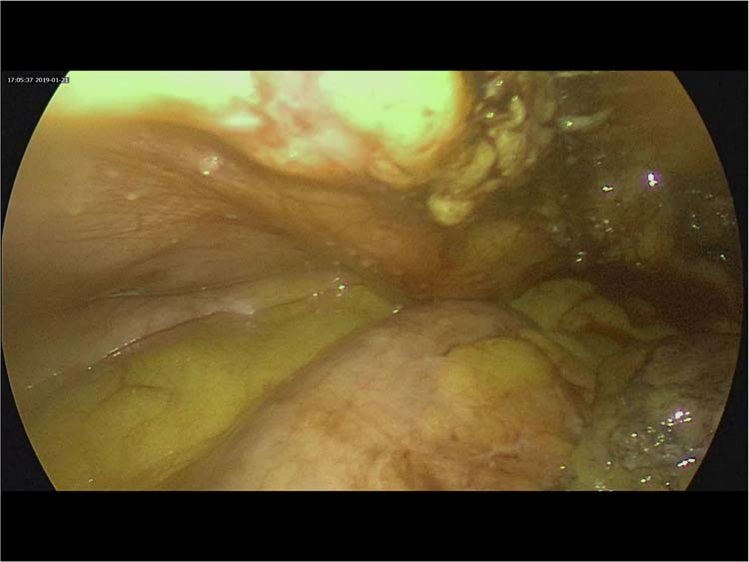
Intra-operative image of peritoneal nodules.

**Figure 5:**
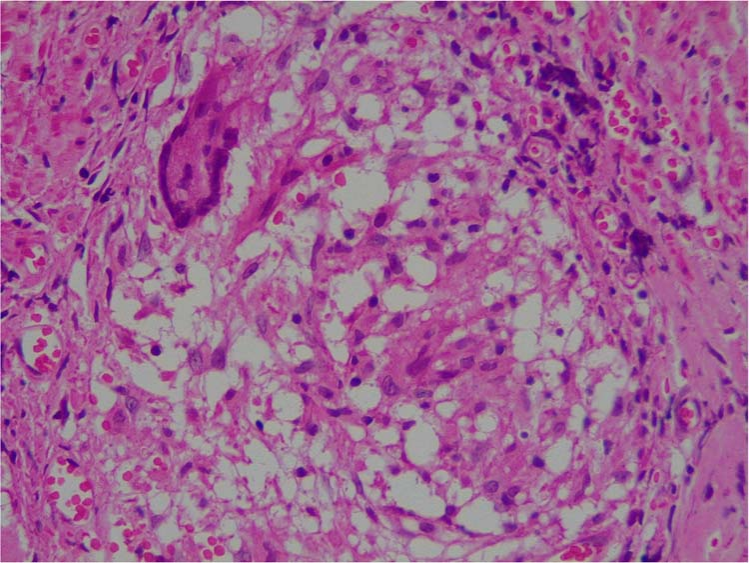
Microscopic image of giant cells.

**Figure 6:**
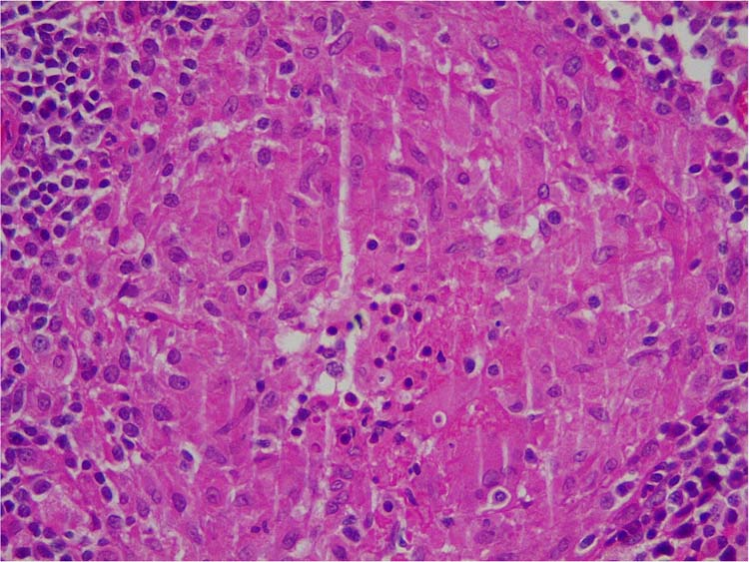
Microscopic image of caseous necrosis.

Intra-operatively, adhesiolysis of dense bowel and omental adhesions were performed. About 3.5-cm appendicular stump was resected (Fig. 3). Micro-nodular peritoneal nodules were noted and resected (Fig. 4). The specimens were sent for a histo-pathology examination. The postoperative period was uneventful with symptomatic relief. The biopsy of the gross specimen of stump appendix described a single white to grey brown soft tissue measuring 3 cm × 2 cm × 0.8 cm with a congested outer surface. The appendicular wall was 0.3 cm thick while the peritoneal nodule was a 2.5 cm × 0.8 cm × 0.5 cm single grey white to yellow soft tissue. Microscopic report of the appendix revealed multiple granuloma composed of epithelioid cells, macrophages, lymphocytes and multinucleated giant cells (Fig. 5). Caseation necrosis was seen in focal areas and with transmural mixed inflammatory cell infiltrate (Fig. 6). The peritoneal nodule showed fatty tissue with infiltration of lymphocytes, plasma cells and neutrophils microscopically.

We started the patient on specific anti-tubercular treatment (ATT), which included rifampicin, isoniazid, pyrazinamide and ethambutol. The patient was discharged on postoperative day 15 with a stable status. The ATT continued for 5 months.

## DISCUSSION

Since Koch’s discovery of the tuberculous bacillus in 1882, improved techniques for tissue sectioning and staining have allowed for a thorough investigation of tuberculosis [9]. Lesions have been discovered in almost every tissue of the body since then. Appendicular TB was first described by Corbin in 1873 [10]. Several cases have since been reported in the literature. Most of them have involved young adults. The average patient age was found to be 25.5 + 8.8 years in a review of the literature spanning ten years between 2010 to 2021 [11] and the same trend can be observed in our case as well. The disease manifests in either primary or secondary form. The primary form could be caused by contact of the appendix with infected intestinal contents, direct hematogenous spread from a distant site such as the lung while the secondary form could be caused by localized ileocecal tuberculosis, retrograde lymphatic spread in the ascending colon or appendiceal serositis/peri appendicitis caused by peritoneal involvement [12].

Appendicular TB can either present as acute appendicitis, or a chronic illness with repeated bouts of right iliac fossa pain, vomiting,and diarrhoea, or as a latent type that is found incidentally [13]. Our report deals with an acute presentation of SA which is another rare postoperative occurrence after appendectomy and was first described by Rose in 1945 [6]. Due to gaps in literature regarding its true incidence and exact causes, diagnosis of the condition remains difficult like appendicular TB. To reduce the risk of developing SA, it is advised that an appendicular stump’s length be less than 0.5 cm [14]. Hence, it is crucial for the operating surgeon to precisely define the appendicular base. The diagnosis is guided by investigations such as an abdominal CT scan and is driven by a high level of clinical suspicion, similar to appendicular TB.

The combination of both these rare conditions makes this case report first of its kind. TB should always be taken into consideration for nonspecific abdominal clinical signs and symptoms because it is a disease that is very common in low- and middle-income countries. The significance of the histological evaluation of appendix is highlighted in this case.

## CONSENT DECLARATION

Informed consent was obtained from the patient for publication of the case and accompanying figures.

## CONFLICT OF INTEREST STATEMENT

None declared.

## FUNDING

None.

## DATA AVAILABILITY

The authors confirm that the data supporting this study are available within the article.

## AUTHORS’ CONTRIBUTIONS

The manuscript was drafted by MM. The images were provided by TM and EJ. All the authors were involved in the care of the patient. The final draft was reviewed and approved by all the authors.
